# The potential role of ischaemia–reperfusion injury in chronic, relapsing diseases such as rheumatoid arthritis, Long COVID, and ME/CFS: evidence, mechanisms, and therapeutic implications

**DOI:** 10.1042/BCJ20220154

**Published:** 2022-08-31

**Authors:** Douglas B. Kell, Etheresia Pretorius

**Affiliations:** 1 Department of Biochemistry and Systems Biology, Institute of Systems, Molecular and Integrative Biology, Faculty of Health and Life Sciences, University of Liverpool, Liverpool L69 7ZB, U.K.; 2 The Novo Nordisk Foundation Centre for Biosustainability, Technical University of Denmark, Kemitorvet 200, 2800 Kgs Lyngby, Denmark; 3 Department of Physiological Sciences, Faculty of Science, Stellenbosch University, Stellenbosch, Private Bag X1 Matieland 7602, South Africa

**Keywords:** amyloid microclots, ischaemia–reperfusion injury, Long COVID

## Abstract

Ischaemia–reperfusion (I–R) injury, initiated via bursts of reactive oxygen species produced during the reoxygenation phase following hypoxia, is well known in a variety of *acute* circumstances. We argue here that I–R injury also underpins elements of the pathology of a variety of *chronic*, inflammatory diseases, including rheumatoid arthritis, ME/CFS and, our chief focus and most proximally, Long COVID. Ischaemia may be initiated via fibrin amyloid microclot blockage of capillaries, for instance as exercise is started; reperfusion is a necessary corollary when it finishes. We rehearse the mechanistic evidence for these occurrences here, in terms of their manifestation as oxidative stress, hyperinflammation, mast cell activation, the production of marker metabolites and related activities. Such microclot-based phenomena can explain both the breathlessness/fatigue and the post-exertional malaise that may be observed in these conditions, as well as many other observables. The recognition of these processes implies, mechanistically, that therapeutic benefit is potentially to be had from antioxidants, from anti-inflammatories, from iron chelators, and via suitable, safe fibrinolytics, and/or anti-clotting agents. We review the considerable existing evidence that is consistent with this, and with the biochemical mechanisms involved.

## Introduction

**Figure 1.**
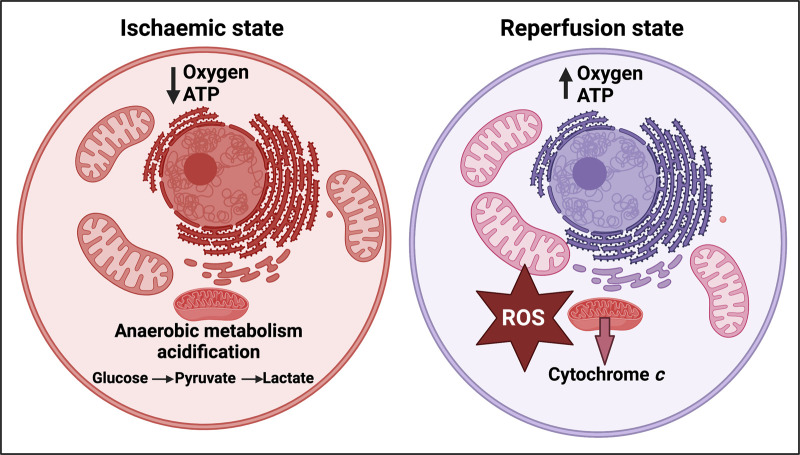
Simplified representation of the initial stages of ischaemia–reperfusion injury (I–R I [7]). Created with BioRender (https://biorender.com/).

If the supply of oxygen to a normally aerobic tissue is restricted (hypoxia, often caused by ischaemia), and then is more or less rapidly restored (‘reperfusion’), that tissue may be damaged. This damage is variously known as ischaemia–reperfusion injury, hypoxia–reperfusion injury, or reoxygenation injury. It is widely observed *acutely* [1], for instance following an acute mycocardial infarction [2–4], stroke [5,6], in emergency medicine [7], and during the *ex vivo* incubation of organs as part of transplant surgery [8–11]. It is considered (see below) that the main mechanisms involve the production, especially during the reperfusion, of various partially reduced reactive oxygen species (ROS) [12,13], not least by an over-reduced mitochondrial respiratory chain that was formed during the hypoxic phase (see Figure 1 for a simplified representation of ischaemia–reperfusion injury (I–R I)). These initiate a variety of other processes such as inflammation that we discuss later.

A considerable number of *chronic* disorders or syndromes seem to occur *following* the acute stages of an infection [14–16], and include rheumatoid arthritis, various forms of more classical chronic fatigue (idiopathic chronic fatigue ICF and myalgic encephalomyelitis/chronic fatigue syndrome ME/CFS), and, most recently, Long COVID [17–23]. They are characterised by a multiplicity of symptoms, that, while seemingly (but not) unrelated, also exhibit two particular features: (i) a variety of manifestations of exceptional levels of fatigue (e.g. [18,24–35] and see below) and (ii) episodic disability [29,36–39], i.e. periods of unwellness that are relatively benign interspersed with periods (‘relapses’ or ‘crashes’) characterised by acute, severe, and often debilitating symptoms. Another striking feature of these diseases is that they predominantly affect women [40–42].

By and large, these diseases (especially ME/CFS and Long COVID) have not been well managed [29,43] because their aetiologies have been uncertain and even their organic (rather than psychological) nature has been questioned. Some of the management practices, such as graded exercise therapy, have even been harmful [43], especially since post-exertional malaise (PEM) (a worsening of symptoms following exertion) is even a hallmark of such diseases [44]. Indeed, we consider (and argue) that periodic ischaemia–reperfusion injury is largely responsible for the relapsing observed.

Thus, the main purpose of this review is to bring together the evidence that a chief cause of such crashes or relapses, that also serves to contribute to the *chronic* nature of such diseases, is probably a kind of chronic *in vivo* hypoxia/reperfusion activity that seems to have been almost completely [45] unremarked. The recognition of this leads to a variety of promising therapeutic opportunities; in most cases, there is already evidence for their benefits. We take such evidence to be evidence for the mechanisms that we propose.

## A multiplicity of symptoms

Depending on the precise scoring, some 10–30% or more of individuals infected with SARS-CoV-2 and exhibiting acute COVID go on to manifest Long COVID [46–54]. This said, Long COVID has over 200 different symptoms, a subset of which (identifiable as subtypes [55]) can occur in any individual [34,56–59], likely with some consequences that may not manifest for years [60,61] or even decades [62]. These include [19,26,27,30,50,56,63–71] breathlessness [72,73], fatigue [17,18,22,25,26,31,34,35,49,74], cardiovascular issues [61,75], chest pain [74], myalgia [17,22,24,25], cognitive dysfunction [26,63,64,74,76–79], innate and cell-mediated immune responses coupled to inflammatory cytokine production [80,81], a variety of coagulopathies [82,83] including fibrin amyloid microclots [84–89], and, in particular, postural tachycardia syndrome (PoTS) [25,26,90,91] and PEM [22,25,31,92].

The great majority of these also occur in both ME/CFS [18,19,43,93–96] (also likely most commonly a post-viral disease [16,19,97,98]) and in rheumatoid arthritis [14,23,99] (where bacterial infection, especially by *Proteus* spp., is strongly implicated [14,100–105]). To retain focus, we do not really discuss the many other chronic, inflammatory diseases for which similar phenomena may be observed (see e.g. [15,106]), though we would comment that ischaemia–reperfusion injury has been suggested to be significant in the vascular disease pre-eclampsia [107,108], that also exhibits many of the other hallmarks (necessarily for a more limited time) of these syndromes [109,110]. Indeed, aspects of COVID-19 bear many similarities to pre-eclampsia in pregnant women [111,112]. To this end, the likely ubiquity of an infectious origin for more or less all chronic inflammatory diseases [15,106] is exemplified by the recent recognition that multiple sclerosis originates with an Epstein–Barr virus (EBV) infection [113–115].

Gene expression levels can vary very widely between different tissues (e.g. [116,117]). Thus, although in acute COVID the SARS-2-CoV virions tend to be most accumulated where their receptors are most prevalent (as were the α and δ-variants in the lungs [118,119]), there is evidence for a wide distribution between tissues [118,120,121], including in Long COVID [19,77,122]. This can help to explain particular differences in symptomology; however, we consider that there are mechanisms (particularly amyloid microclot formation [85] and, as introduced here, regular ischaemia–reperfusion injury) that are also general enough to help to understand the unusual breadth of the pathology of Long COVID.

## A systems approach

**Figure 2.**
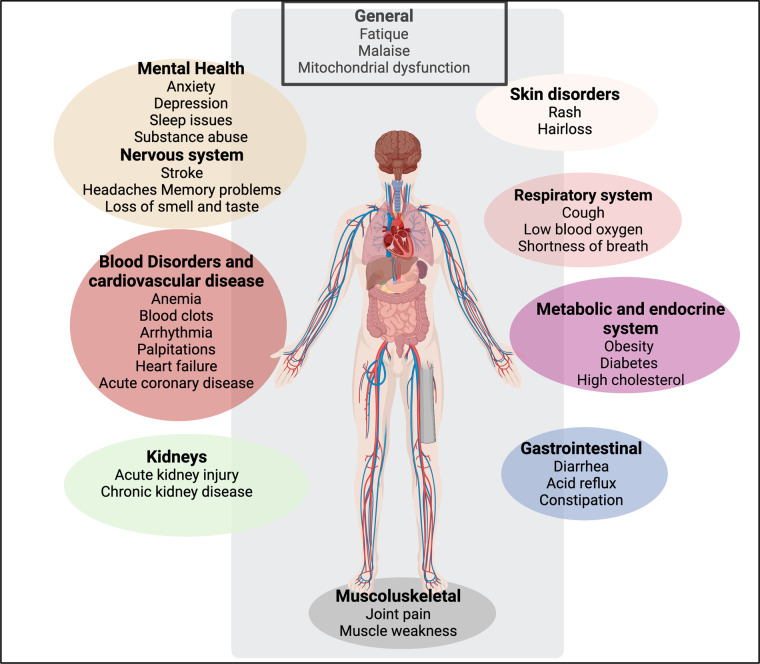
Systems that are likely to be affected by, and contribute to, chronic, inflammatory diseases. Created with BioRender (https://biorender.com/).

**Figure 3.**
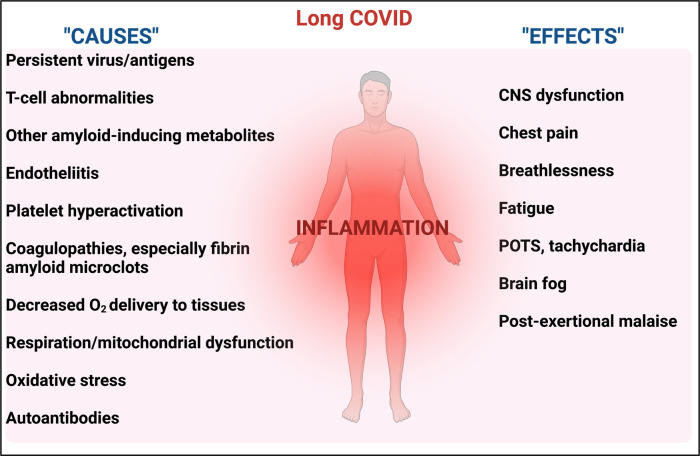
Some causes and effects of inflammation. In many cases there is positive feedback in which A causes B and B causes A (e.g. inflammation ßà endotheliitis, inflammation ßà coagulopathies). Created with BioRender (https://biorender.com/).

Given the complexity of the potential actors and symptoms, and the fact that in one sense everything is connected to everything else, often with complex feedback loops, the task of the systems biologist is to identify, and ultimately to quantify, the pathways and elements most strongly contributing to the phenomena of interest. This will initially take the form of qualitative network diagrams [123–126]. To this end, Figure 2 indicates some of the features that we consider likely to contribute to these chronic, inflammatory diseases, along with an attempt (Figure 3) to indicate some evident causal relationships.

Many the following sections summarise the mechanistic evidence for these phenomena. Given the heterogeneity of symptoms alluded to above, it is clear that some pathways will be more significant than others in particular individuals, so this is necessarily to be seen as a high-level view. In particular, to understand how one state of relatively benign conditions can morph into another of much greater severity, it is to be recognised that normally an internal or external trigger of some kind is necessary. This could be hormonal (e.g. the female menstrual cycle) or more or less any kind of stress or trauma. Our focus here is on the involvement of a kind of chronic ischaemia–reperfusion injury in these processes, and the role of fibrin amyloid microclots therein.

## Ischaemia–reperfusion injury and ROS production

Under normal circumstances, the reduction in dioxygen by the mitochondrial respiratory chain is a four-electron reduction (leading to water), occurring at cytochrome *c* oxidase. If the respiratory chain is over-reduced, however, as a result of hypoxia or the ischaemia that causes it, O_2_ when readmitted (‘reperfusion’) can undergo a two-electron reduction at the level of cytochrome *b* (complex III) forming peroxide, or a one-electron reduction at complex I forming superoxide ${\text{O}}_{2}\cdot^{-}$. A variety of other oxygen-reducing enzymes can also lead directly to the production of such ‘reduced’ forms of dioxygen *in vivo* (e.g. [1,127–129]), with H_2_O_2_ from xanthine oxidase being especially implicated in ischaemia/reperfusion injury (e.g. [128,130–135]).

Superoxide dismutase [136] can serve to equilibrate superoxide and peroxide:

1 $$\text{2 }{\text{O}}_{2}\cdot^{-}\;+\text{2}{\text{H}}^{+}\to {\text{H}}_{2}{\text{O}}_{2}+{\text{O}}_{2}$$

As reviewed in detail previously [106], although classed as ‘reactive oxygen species’, neither peroxide nor superoxide are normally excessively toxic at low concentrations (indeed they can be used as signalling molecules); what does cause major toxicity is their presence at higher levels and in addition the reaction of hydrogen peroxide with (free or poorly liganded) Fe(II) in the Fenton reaction [137], leading to the very reactive and damaging hydroxyl radical (OH⋅)

2 $$\text{Fe}(\text{II})+{\text{H}}_{2}{\text{O}}_{2}\to \text{Fe}(\text{III})+\text{O}{\text{H}}^{-}\;+OH\cdot$$

Superoxide can also react with ferric iron in the Haber–Weiss reaction [138,139] to produce Fe(II) again, thereby effecting redox cycling:

3 $${\text{O}}_{2}\cdot^{-}+\text{Fe}(\text{III})\;\to {\text{O}}_{2}+\text{Fe}(\text{II})$$

It is to be stressed that the combination of these two reactions means that unliganded iron can act *catalytically* to produce hydroxyl radicals.

**Figure 4.**
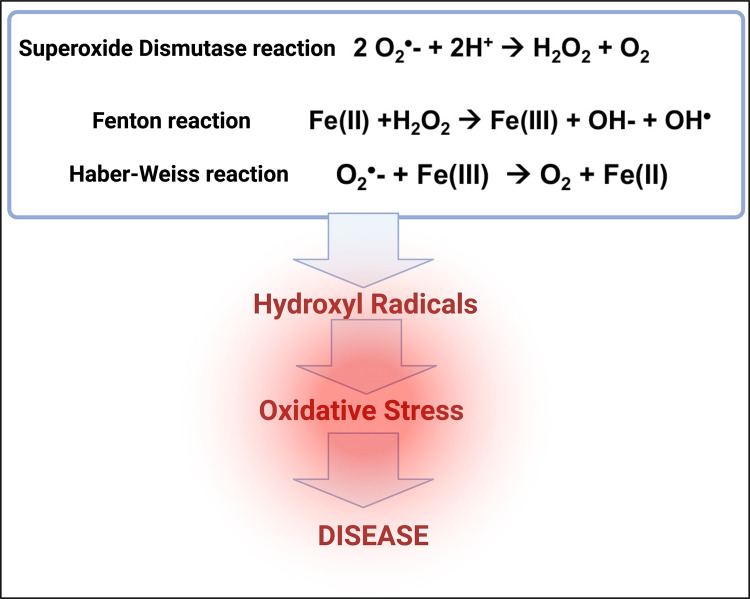
Some of the chemical reactions contributing to oxidative stress and disease. Created with BioRender (https://biorender.com/).

Hydroxyl radicals are exceptionally reactive, and react within nanoseconds with anything that is nearby. Thus it is they that are especially responsible for all the trouble connected with oxidative stress (Figure 4).

Note that ascorbate can replace ${\text{O}}_{2}\cdot^{-}$ within the cell for reducing the Fe(III) to Fe(II) [140]. Thus, although ascorbate is ‘reducing’ and an ‘antioxidant’, its reaction with O_2_, especially when catalysed by Fe(II), produces superoxide and thence OH⋅ radicals that may be *pro*-oxidant [141]. Indeed, a variety of clinical trials using ascorbate in diseases considered to be accompanied by oxidative stress have found that it is actually less good than was the control/placebo in terms of all-cause mortality [142,143].

## The importance of liganding free ‘iron’

This was discussed in much more detail previously [106] (see also [15]), so only a brief summary is given here. Specifically, while iron is familiar as normally existing mainly in Fe(II) or Fe(III) states, this describes only part of the picture, since the Fe atom contains six potentially ligandable sites (two polar, four equatorial) that also affect its reactivity, and only when all six sites are occupied (liganded) is its reactivity controlled. Liganding say four sites actually *increases* its reactivity, and for instance the reduction by ascorbate of Fe–EDTA complexes (that ligand only the equatorial sites) is in fact a potent means of producing hydroxyl radicals in the laboratory [144–147]. Thus it is vital to ensure that free iron, and especially poorly liganded iron, is absent [148]. Note, in this context, that cell death is accompanied by the release of ferritin, and that this ferritin (which should not normally be in plasma at all) liberates free iron when it enters the bloodstream [149].

## Dormancy in bacteria and viruses

Although textbooks of microbiology focus on the kinetics and behaviour of microbes when they are (or have recently been) actively growing, this state is not at all the norm in nature. If an organism can grow it will do so, but if it lacks a necessary nutrient or signalling molecule it cannot (by definition). Unsurprisingly, evolution long ago selected against organisms that simply die when unable to grow, and instead selected for starvation survival [150–153] that required that they entered a state of dormancy [154,155], from which they must in time become resuscitable [156–159]. In clinical microbiology (where large segments of the human population harbour dormant forms of *Mycobacterium tuberculosis* [160,161] and/or *Helicobacter pylori* [162,163] without manifesting overt infection), such dormant forms are often referred to as ‘persisters’ [164–174].

**Figure 5.**
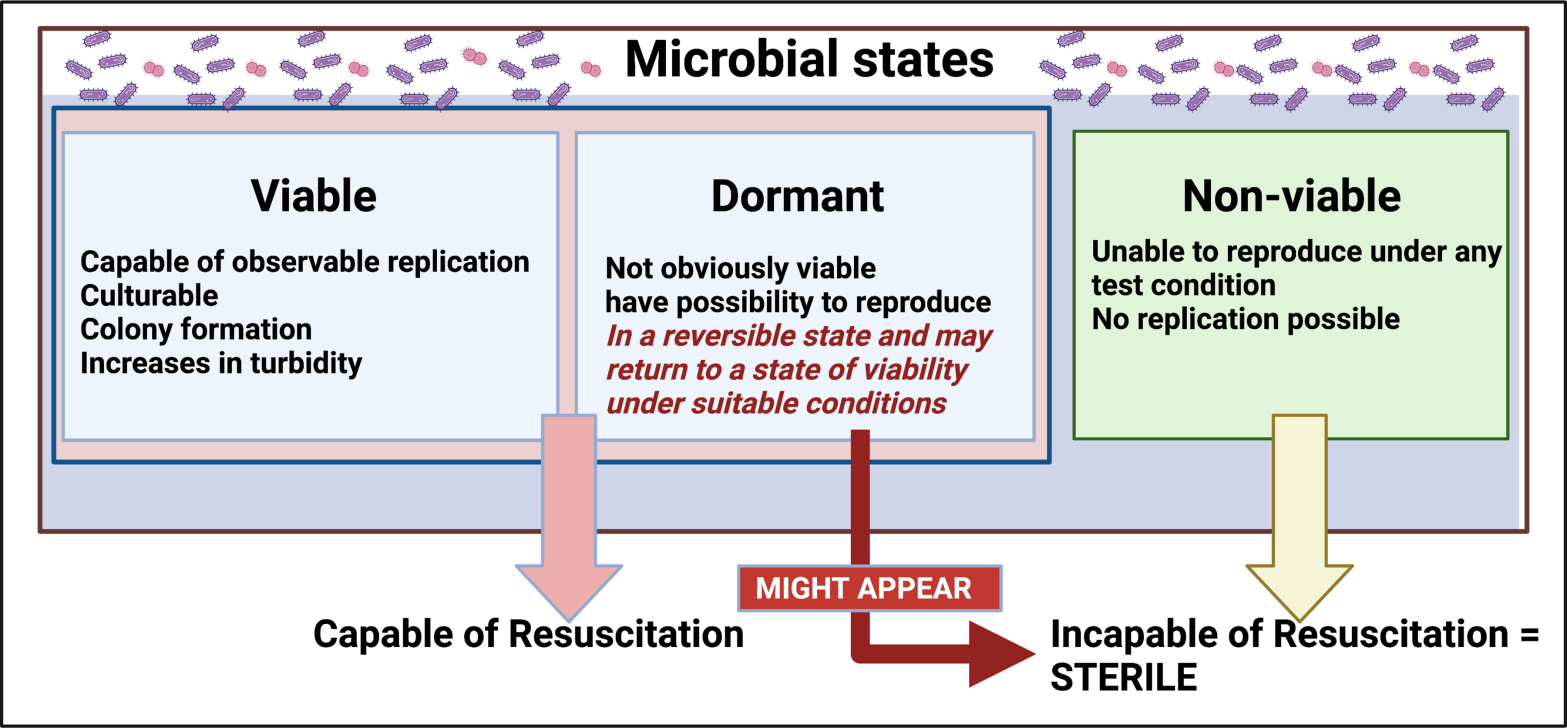
Microbial states and their viability and culturability, adapted from [15]. Created with BioRender (https://biorender.com/).

Dormant bacteria represent a conundrum for classical bacteriology, since they do not obey the classical Koch's postulates that classically allow one to state that organism X causes disease Y [175–182]; specifically, they do not necessarily adopt replicating forms, that might be assessed as axenic colonies on petri dishes. Notwithstanding claims of reagent contamination, the presence of dormant (non-replicating) bacteria also underpins the existence of (often resuscitable) microbial cells in blood [183–193] and tissues (e.g. [109,110,194]) that are normally considered to be necessarily sterile, where they may also be detected by molecular methods or using imaging techniques. In general terms, the invasion of host cells by microbes (‘intracellular pathogens’) that are known to lie in a dormant or quiescent state and reactivate at later times is actually a commonplace (e.g. [195–222]), so should not, in fact, be perceived as ‘controversial’ at all (Figure 5).

Viruses, especially herpes viruses, but also enteroviruses [223], can also remain latent for years [224–226], a well-known example being the activation of herpes zoster (manifesting as shingles) decades after a typically childhood infection with chickenpox [227]. Their reactivation has also been strongly implicated in Alzheimer's disease [228–231]. The prevalence of latent herpesviruses such as cytomegalovirus may be as great as 90% [232,233]. Most pertinently, there are strong indications for a role of periodic viral (and/or bacterial) reactivation in both ME/CFS [234] and Long COVID [16,19,22,43,47,220,235–239].

## Triggers of viral reactivation

A chief means by which viruses are suppressed, or maintained in a latent state, involves the interferon system (e.g. [240–246]). For present purposes, it is not necessary to go into all the molecular details; sufficient here is to recognise that infection with SARS-CoV-2 (as with certain other viruses [242,247–249]) can effectively lower the normal interferon responses [250], and it is this that effectively unleashes existing latent viruses. Vaccination can sometimes elicit a similar effect [251–253].

## Triggers of bacterial reactivation

With the possible exception of *Borrelia burgdorferi* (the causative agent of Lyme disease) [254], all microbes need a source of iron. Normally, within human hosts, free iron is strictly regulated and is not available to assist the growth of pathogens [15,255–263]. This is (in part) why typical pathogens cannot replicate *in vivo*, why higher iron levels correlate with infection [15], and why genes encoding iron uptake mechanisms are virulence genes [264,265]. Thus, any kind of trauma that leads to cell death can liberate free iron [15,266], which then initiate replication (and the potential shedding of inflammagens such as lipopolysaccharide (LPS) [15,267] and lipoteichoic acid [268]).

## Endotheliitis

It is by now well established that endotheliopathies, commonly measured using flow-mediated dilation [269,270], are of great significance in the pathological response of hosts to SARS-CoV-2 infection [271–284]. They are initially caused by the viral infection, but likely both lead to and are caused by the coagulopathies and strong inflammatory responses that characterise the disease.

## Coagulopathies

The term coagulopathies is used to describe any kind of dysregulation of the blood coagulation system. Coagulopathies [82,285–302] are a hallmark of both acute COVID [82,84,86,303–319] and Long COVID [88,89,320], and spike protein may be activated by clotting factors [321]. Acute COVID-19 is associated with both a hypercoagulable state and with bleeding; the resolution of the apparent paradox is temporal [82] since the hypercoagulation can use up elements such as von Willebrand factor (VWF) that are then insufficient for normal coagulation to occur. Hypercoagulation can be caused by any number of traumas or shocks to the system, from the more obviously acute kind of trauma [322–324], to the presence of molecules such as free iron [325–330], bacterial LPS [267,268,331–333], and lipoteichoic acid [268], or, most pertinently, the S1 spike protein of SARS-CoV-2 [84]. Coagulopathies are also a feature of rheumatoid arthritis [14,334–337] and indeed all kinds of inflammatory diseases [326,338–345], including of course COVID-19 [346–349] and ME/CFS [350–352]. However, although these links are well established, there is an important twist that makes the kind of coagulation we are talking about *anomalous* because it leads to fibrin *amyloid-containing* clots that are more resistant than usual to fibrinolysis.

## Fibrin amyloid microclots triggered by bacterial and viral components

Since we are not normally talking about overt infection here, in which large numbers of proliferating organisms overwhelm the host and create sepsis [353], we need a mechanism (or set of mechanisms) by which more-or-less tiny amounts of viral or bacterial product can amplify their effects. In this case, we believe that the clotting of blood into an anomalous, amyloid-type form, is a major contributor [85]. The terminal stages of blood clotting involve the self-organised polymerisation of fibrinogen (a protein of dimensions ca 5 × 45 nm) into fibrin fibrils of great length, and diameters of say 50–100 nm. What we discovered was that highly substoichiometric amounts of the Gram-negative bacterial cell wall component LPS , viz 1 molecule LPS per 100 000 000 fibrinogen molecules, could induce the clotting to be into a thermodynamically more stable, protease-resistant amyloid form [354–356]. This could also be effected by the Gram-positive equivalent, lipoteichoic acid [268], and, importantly for present purposes, by the spike protein of SARS-CoV-2 [84,85,87–89,306,357]. (We have not yet tested surface antigens from other viruses.) Because the conversion of normal forms of proteins to their amyloid equivalents, with no change in primary sequence, is often triggered by the amyloid form itself, much as with prions [355,358–361], it is unsurprising that there is cross-reactivity between amyloid types [362–365], and amyloid deposits of various kinds are also a hallmark of SARS-CoV-2 in COVID-19 [85,87,89,306,366–368], as well as with other viruses [369] and diseases [364]. One feature of the amyloid form is that by representing a conformation different from that of normal fibrin it necessarily displays novel epitopes, which can of course select for the production of autoantibodies; the fibrinaloid clots also trap a variety of other molecules, including plasmin-inhibiting agents [88] that mechanistically may contribute to their stability. However, probably the most important *consequence* of the resistance of these fibrin amyloid or ‘fibrinaloid’ [85] microclots to fibrinolysis is that they are able to pass into microcapillaries and block them up [370]. Such fibrinaloid microclots have now recently been observed in the plasma of ME/CFS, at levels some 10-fold those of healthy controls [352].

We also note that *in vitro* studies have shown that various other molecules can induce fibrin amyloid formation (referred to in our early literature as ‘dense matted deposits’); these include oestrogen [371] (which we consider also reflects the prevalence of these diseases in women [17,40,72] and variations in the severity of Long COVID during the female monthly cycle) and unliganded iron [326,330,372,373]. In contrast, magnesium ions are protective against fibrin amyloid microclotting [374]. We recognise, however, that men also produce oestrogens, so it is not a straightforward correlation, and many elements might underlie the significant bias towards women in these and other chronic, inflammatory diseases.

## Evidence for capillary blockage from venous O_2_ levels

**Figure 6.**
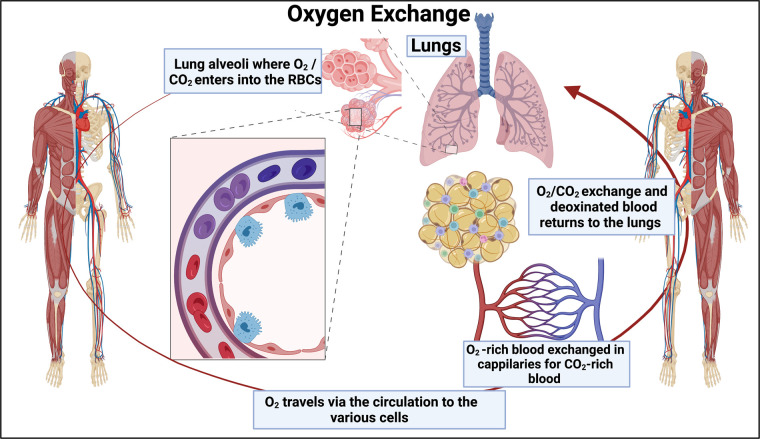
Simplified diagram of oxygen exchange, showing arteries (red) and veins (blue) linked by capillaries. Created with BioRender (https://biorender.com/).

Tissue hypoxia can be caused by a variety of mechanisms, including poor O_2_ uptake in the first place or poor exchange with tissues. Similarly, mitochondrial respiration, whose control is distributed across many steps [375], may also be limited by a variety of causes, including the number of mitochondria and, of course, the actual availability of O_2_. To rehearse, arteries take O_2_-poor blood from the heart to the lungs where it is oxygenated, capillaries distribute it throughout tissues, and veins return the less-oxygenated blood to the heart (Figure 6). Nowadays the venous blood saturation is measured via a central venous catheter in the superior vena cava via the jugular of the subclavian vein, rather than in the pulmonary artery as previously [376]. It reflects both O_2_ transport to the tissues and usage by them [377], so while a low value is indicative of problems a normal value may not entirely reflect them [376]. Specifically, the evidence that in many of these diseases tissue hypoxia is a major cause of problems due to the capillaries not doing their job properly comes from the measurement of central or mixed venous (vs arterial O_2_) saturation [378,379]. In one study [378], central venous oxygen saturation (ScvO_2_) was 66% for healthy controls, 33% for acute COVID-19 survivors, and 18% for those who died from COVID-19. We note that conventional fingertip-type pulse oximeters, while reasonably accurate, mostly estimate arterial O_2_ levels [380], so would not necessarily pick up this kind of phenomenon; indeed this may account for the discordance [381,382] between pulse oximetry and arterial blood gas measurements. Cerebral oximetry [383] may provide a truer assessment if non-invasive measures are desired.

One typical consequence of a blockage of a flexible pipe is that it swells. While it is not so easy to detect this, one organ where this is indeed visible is the eye. Consistent with capillary blockage, the diameters of blood vessels in the eye increase during COVID-19 [384–386]. Specifically, the likelihood of retinal microvasculopathy in subjects with COVID-19 was massively higher compared with that of controls (with an odds ratio and 95% confidence interval of 8.86 and 2.54–27.53, respectively [387]). In some cases, even ocular vein thrombosis has been observed [388].

## Heart-rate variability

If — as argued here — capillary blockage induced by fibrin amyloid microclots is important, especially as output demand is increased, it is reasonable that heart-rate variability might be increased in individuals suffering from Long COVID. This very much turns out to be the case [389–391], and is presumably related to postural orthostatic tachycardia syndrome (POTS) [392], another common symptom of Long COVID [21,90,393–395] with likely a similar cause. Specifically, as well as more complex physiological contributions, low O_2_ is a standard signal (as occurs during exercise) for the heart to beat faster (e.g. [396]); in the cases of interest here, we simply posit that this occurs at far lower levels of exercise effort because of the impaired ability to deliver O_2_ to tissues.

## Size of fibrin amyloid microclots and capillary diameters

**Figure 7.**
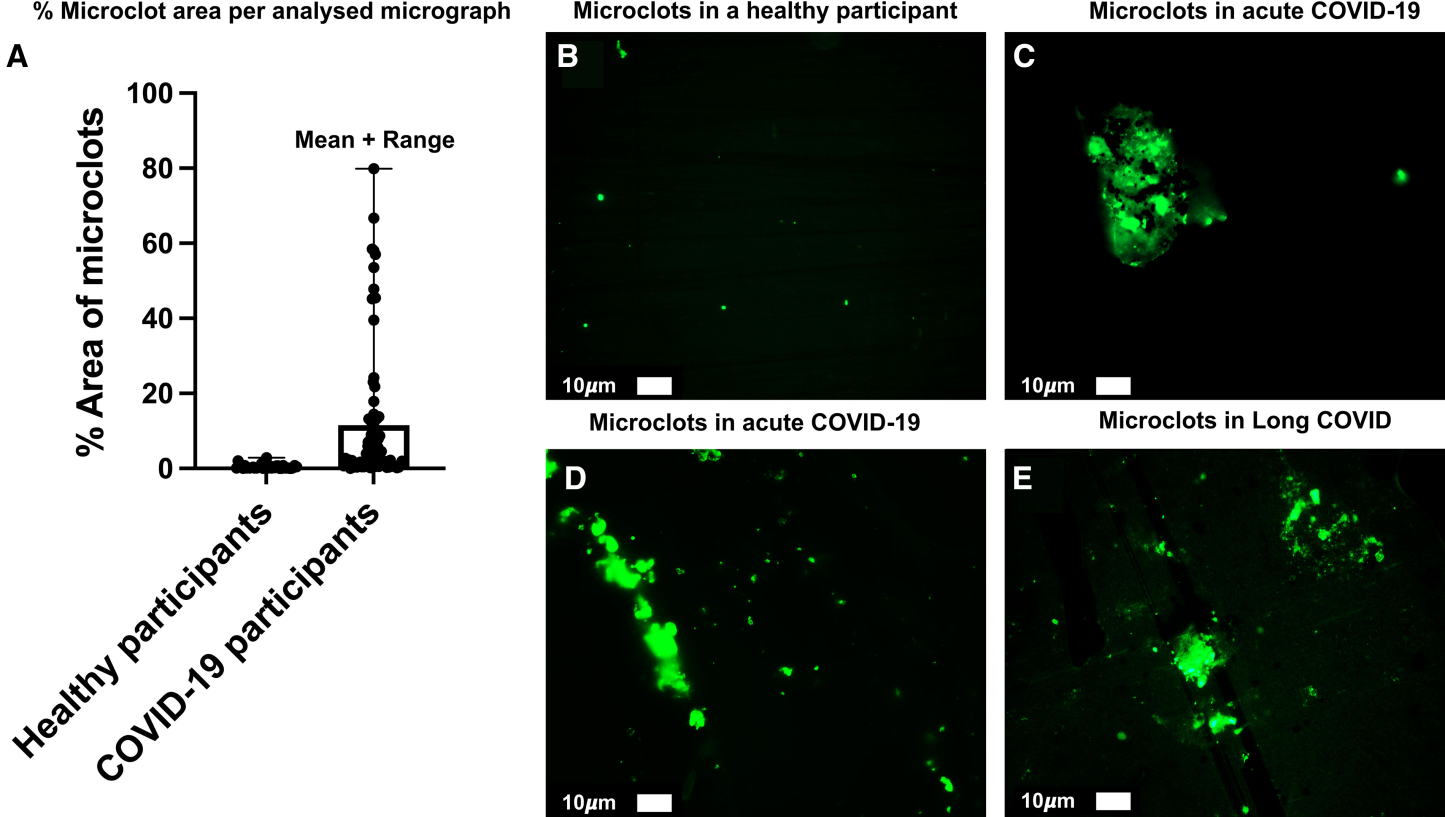
(**A**) % area distribution of microclots in plasma from participants with acute COVID-19 (taken from raw data as in [87]). (**B**) Representative micrograph of microclots in plasma from a healthy individual. (**C**,**D)** Representative micrographs of microclots in plasma from acute COVID-19 participants (taken from raw data as in [87]). (**E**) Representative micrograph of microclots in plasma from participants with Long COVID (taken from raw data in [422]).

Most capillaries (depending on the individual under study [397]) are just 5–10 μm in diameter [398–406], which means that erythrocytes, with a discoid shape of 6–8 μm diameter × 2–2.5 μm [407], largely pass through them in single file (and their viscosity is here at a minimum [408]). Others are more like 15–50 μm diameter [409,410]. (There is thus a size discrepancy between the diameter of the white blood cells (6–8 μm or more) and that of the smallest capillaries (∼5.5 μm) and this size discrepancy forces the white blood cells, to deform in order to transit the capillary bed [411].) The transition into the tissue over the blood vessel walls will occur in the response to a biologically harmful stimulus (e.g. inflammation or infection) [412]. This white blood cell migration process is called transendothelial migration (TEM) or diapedesis. The fibrin amyloid microclots that we discovered are commonly 5 to even 200 μm in diameter, so it is not surprising that they can block up capillaries. See Figure 7 for representative examples of microclots in healthy participants (exposed to neither SARS-CoV-2 nor vaccine), those found in acute COVID-19 and in Long COVID. Also, note the % area distribution of microclots in acute COVID (data published in [87]). In addition, the erythrocytes themselves lose deformability in these kinds of disease [413]. Improved imaging methods coupled to advanced computer vision algorithms for assessing such capillary diameters (e.g. [398,399,409,414–421]) and their relation to blood flow represent important areas of research.

Two other major points pertain to this question about the relationship between the size of capillaries and the likelihood of insoluble circulating material clogging them: the first is that if microclots of whatever composition are ‘already’ trapped in capillaries for a greater or lesser time they will not be observable in standard venous plasma samples, and so these measurements may underestimate the problem. The second is that this general mechanism of capillary blockage is likely true for other kinds of debris including those from endothelial cell damage; to this end, it is worth recognising that the presence of extracellular vesicles is part of many chronic diseases (e.g. [423–427]), including rheumatoid arthritis [428–433]. In particular, extracellular vesicles are seen as a highly discriminating accompaniment to ME/CFS [434], and are being observed in both acute [435] and Long COVID-19 [436] patients.

## Erythrocyte (RBC) deformability and eryptosis

The size, shape, and deformability of erythrocytes contributes strongly to their function, and there is evidence that these too are abnormal in the diseases of present interest, viz rheumatoid arthritis, ME/CFS, acute and Long COVID. The extent to which these are caused via fibrin amyloid microclots or by other means (such as reaction with ROSs [437,438]) is unknown, but this is also likely to contribute to the oxidative stress observed. In particular, RBCs from patients with ME/CFS were significantly non-discocytic [439] and stiffer [413] than those from healthy controls. Differences are also found for erythrocyte sedimentation rates (ESR) [413,440] (as it was in the case of delayed cerebral ischaemia following subarachnoid haemorrhage [441]) and also for the easily measured red cell distribution width (RDW) [442]. ESR values are changed in Long COVID [443], while RDW is raised substantially (and increasingly with severity) in acute COVID [444–448]. We are not aware of studies of RDW and Long COVID, but the ease of measurement of this variable implies that much more use might be made of it.

**Figure 8.**
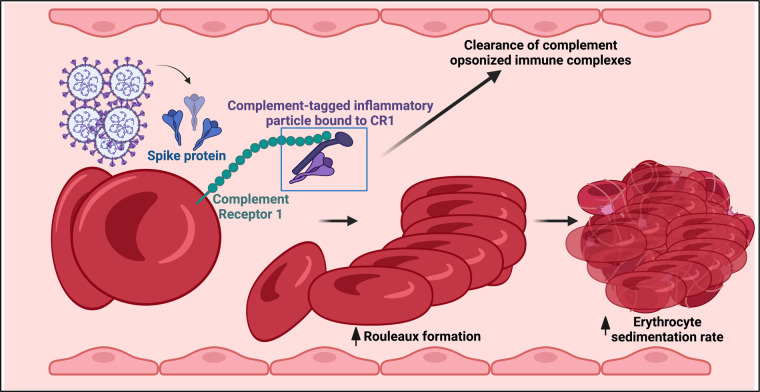
Immune complex formation on erythrocytes during COVID-19 and Long COVID: a hypothesis. Created with BioRender (https://biorender.com/).

It was also found that in acute COVID-19 infection, complement deposition on erythrocytes occurs [449]. Erythrocytes have complement receptor 1 (CR1) (also known as C3b/C4b receptor or CD35) on their membranes and this binds complement activation products C3b and iC3b [449] and C4d [450]. The complement system promotes clearance of pathogens but pathological complement activation may cause microvascular thrombosis. In COVID-19 ICU patients, a reduced CR1 erythrocyte density was observed. Furthermore, deposits of C4 fragments on erythrocytes and virus spikes or C3 on erythrocytes among COVID-19 patients may be of significance to the clearance of immune complex or complement fragment-coated cell debris during COVID-19 infection [450] (see Figure 8). All of these aspects can contribute to impaired blood flow and hence lowered tissue oxygenation.

An extreme endpoint of oxidative stress-inducing erythrocyte stiffness and altered morphology is eryptosis [451–455], in which erythrocytes undergo a specific form of programmed cell death. However, since it can also be induced by a variety of drugs sometimes used to treat the relevant diseases (e.g. [456]), it may not be the best biomarker for diagnosing them precisely. Erythrocyte pathology is nonetheless a clear indicator of problems with microcirculation [453,457–459], and as such speaks clearly to our proposals that an inadequate microcirculation, leading to I–R injury, lies at the heart of these diseases.

## Thromboses

While amyloid microclots in capillaries have been our focus, we should recognise that anything disposing to their formation might, statistically, tend to lead to the formation of larger clots. When the blockage by such a clot of a larger blood vessel such as a vein is effectively complete, one consequence is a venous thromboembolism (VTE), including pulmonary embolism (PE) and deep vein thrombosis (DVT). It is, therefore, worth enquiring as to whether the likelihood of these is raised as part of the diseases we are considering. The answer is that individuals with rheumatoid arthritis [460–467] and acute [287,468–479] and Long COVID [370,480,481] are indeed significantly more prone to such VTEs. The extent to which these thrombi are amyloid in character seems not to have been investigated, and there is also an intriguing link with MAST cell contents [482]. Here, we should also mention the prevalence of microthrombi and VTEs as part of sepsis (e.g. [353,483–485]), and the similarities between some of the symptoms observed in Long COVID or ME/CFS and what is coming to be recognised [486–489] as post-sepsis syndrome. These events provide further mechanistic evidence for the kinds of microcapillary blockage we believe to be caused by fibrinaloid microclots.

## Aneurysms

An aneurysm is a bulge in a blood vessel caused by a weakness in the blood vessel wall, commonly where it branches. Clearly, the blockage of a blood vessel by a microclot is likely to exert pressure on the vessel's walls, potentially weakening it, and so it is reasonable that aneurysms might be expected to accompany diseases involving microclots. This turns out to be the case in rheumatoid arthritis [490], acute COVID [491,492] (which bears similarities to Kawasaki disease [493]) and Long COVID [17]. The association with ME/chronic fatigue syndrome is seemingly weaker, and is usually seen as an association of fatigue, for example, driven by a subarachnoid haemorrhage [494,495].

## COVID toes

Chilblains (pernio) commonly occur when the extremities get cold (restricting blood flow — ischaemia) followed by a period of rewarming (with reperfusion) leading to what might be seen as almost a classical and observable form of ischaemia–reperfusion injury. An unusual and striking feature that has been observed in both acute [496–502] and Long COVID [503–505] is just such a set of ‘chilblain-like lesions’ commonly known as ‘COVID toes’. They seem to reflect thromboembolic events, as well as a strong interferon response [506], and may represent (and certainly provide evidence for) one of the most visible sequelae (both macroscopically and via capillaroscopy [507–509]) of the kinds of ischaemia–reperfusion injury that we are positing here.

## Evidence of hypoxia leading to oxidative stress

There is no doubt that hypoxia leading to ROS is the cause of ‘oxidative stress’ [510]. Hypoxia can have a variety of causes [511], and was of course one of the chief hallmarks of early variants of SARS-CoV-2 in acute COVID-19 [370,512–521], and it is reasonable that this is in part due to impaired capillary flow [370,522]. It can only be modelled if perfusion defects are included [523]. It is similarly a characteristic of Long COVID [17] and also, perhaps less well known, of rheumatoid arthritis [524]. Rheumatoid arthritis is also correlated with ischaemia as judged by a comorbidity with ischaemic heart disease [525], and indeed with VTE [461] and anomalous amyloid clotting [14,336,526]. Many positron emission tomography (PET) probes for detecting hypoxia *in vivo* do exist [527], and of course for research purposes one may use suitable optical probes [528] or the assessment of gene expression profiles as modulated by the transcription factor hypoxia-inducible factor (HIF-1) [529–531]. Such evidence for hypoxia is an important part of the narrative we bring together here.

## Oxidative stress resulting from ischaemia–reperfusion injury

Oxidative stress refers to an imbalance between the rate of production of reactive oxygen and nitrogen species (many of which are free radicals) and their elimination via antioxidants (e.g. [106,532–545]). As discussed above, any ischaemia or hypoxia followed by a return to normoxia runs the risk of ischaemia–reperfusion injury, since ROSs are the inevitable consequence. This manifests as a combination of oxidative stress, inflammation, and (synonymously) inflammatory cytokine production. This is almost so well known that it needs no rehearsal, so we merely give a few reviews as they pertain to rheumatoid arthritis [14], to ME/CFS [16,18,19,29,43,44,97,546–548], and to Long COVID [17,19,25,27]. For us, the key question is: how is this most obviously manifest in something relatively easily measurable (that thus provides evidence for them)?

## Lactate as a measure of ischaemia

If the supply of O_2_ to tissues is inadequate, cells must rely on non-respiratory reactions for their ATP formation, meaning that lactate is likely to accumulate. To this end, elevated lactate accumulation has indeed been observed in rheumatoid arthritis [549–551], in ME/CFS [552–557], and in acute [558–561] and long [562] COVID.

## Measurable hallmarks or biomarkers of ischaemia–reperfusion injury

As rehearsed above, and in much more detail previously [15,106], while peroxide and superoxide are intermediates, and at high levels are cytotoxic, it is also the hydroxyl radical that causes many or most of the manifestations of oxidative stress and, in particular, of ischaemia–reperfusion injury. Because it is so reactive, it is not observable directly; however, the products of the effective reaction of ROSs with proteins (nitrotyrosine [563–567]), lipids (malondialdehyde [568,569]), and DNA (8-oxoguanine [570–574]) are. Consequently, if we wish to know that I–R injury is a contributor to the kinds of syndromes we are discussing here, we ‘simply’ need to check whether they manifest these oxidative biomarkers. 8-isoprostane is another, and it (and the related 15-isoprostane [575]) is raised in rheumatoid arthritis [576,577], ME/CFS [546], and COVID-19 [578–581]. Malondialdehyde and related compounds from lipid peroxidation are also easily measured in the ‘thiobarbituric acid reactive substances’ (TBARS) assay. Note, however, that being ‘endpoint assays’ they can measure determinands that can accumulate, but since the degradation rate of such molecules is normally unknown they cannot alone be used to determine the *recency* of any oxidative stress.

This said, there is plenty of evidence for the presence of nitrotyrosine in acute COVID [582,583], rheumatoid arthritis [584–587], and ME/CFS [588]. It does not yet seem to have been looked for in Long COVID but has been anticipated [583]. TBARS levels are also raised in rheumatoid arthritis [589–591], acute COVID [580], ME/CFS [592–595], and idiopathic chronic fatigue [94], and are similarly anticipated in Long COVID [547]. These measurements provide powerful evidence for the role of ischaemia–reperfusion phenomena in ME/CFS and Long COVID.

## Post-exertional malaise

‘Fatigue’ in syndromes such as ME/CFS and Long COVID is commonly used to refer to an inability at a given moment to perform what would normally be seen as simple and very light mechanical, exercise or even cognitive tasks. In contrast, PEM refers to a state that *follows* such activities, commonly 24–48 h later, in which many of the symptoms of ME/CFS are exacerbated, and may be accompanied by burning sensations. Such events are often referred to as ‘crashes’. According to the ideas developed here, PEM is precisely a consequence of ischaemia–reperfusion injury, because the kinetics of cell death are such that this does not happen instantaneously but follow the flood of ROS contingent on the reperfusion events.

## Inflammation

There is a certain circularity in the definition of inflammation, since inflammatory cytokines (such as IL-1β, IL-6, and TNF-α) are those that rise during inflammation, while inflammation is decreed when inflammatory cytokines are raised. Many are controlled by the transcription factors NF-κB [596] and Nrf2 [597,598], both of which are sensitive to redox state/oxidative stress [599–606], thereby providing a straightforward link between hypoxia, oxidative stress, and inflammation [607–609]. However, most measurements of these transcription factors are performed on ‘static’ samples, while it is their oscillatory nature that controls gene expression [610–614]. Consequently, beyond the qualitative statement of a linkage between these transcription factors and redox state (i.e. oxidative stress) the interpretation of most available data is not straightforward. What is also well established, however (e.g. [9,615–620]), is that inflammation is absolutely a consequence of ischaemia–reperfusion injury and ROS production, and vice versa, providing a ‘vicious cycle’.

## Mast cell activation

**Figure 9.**
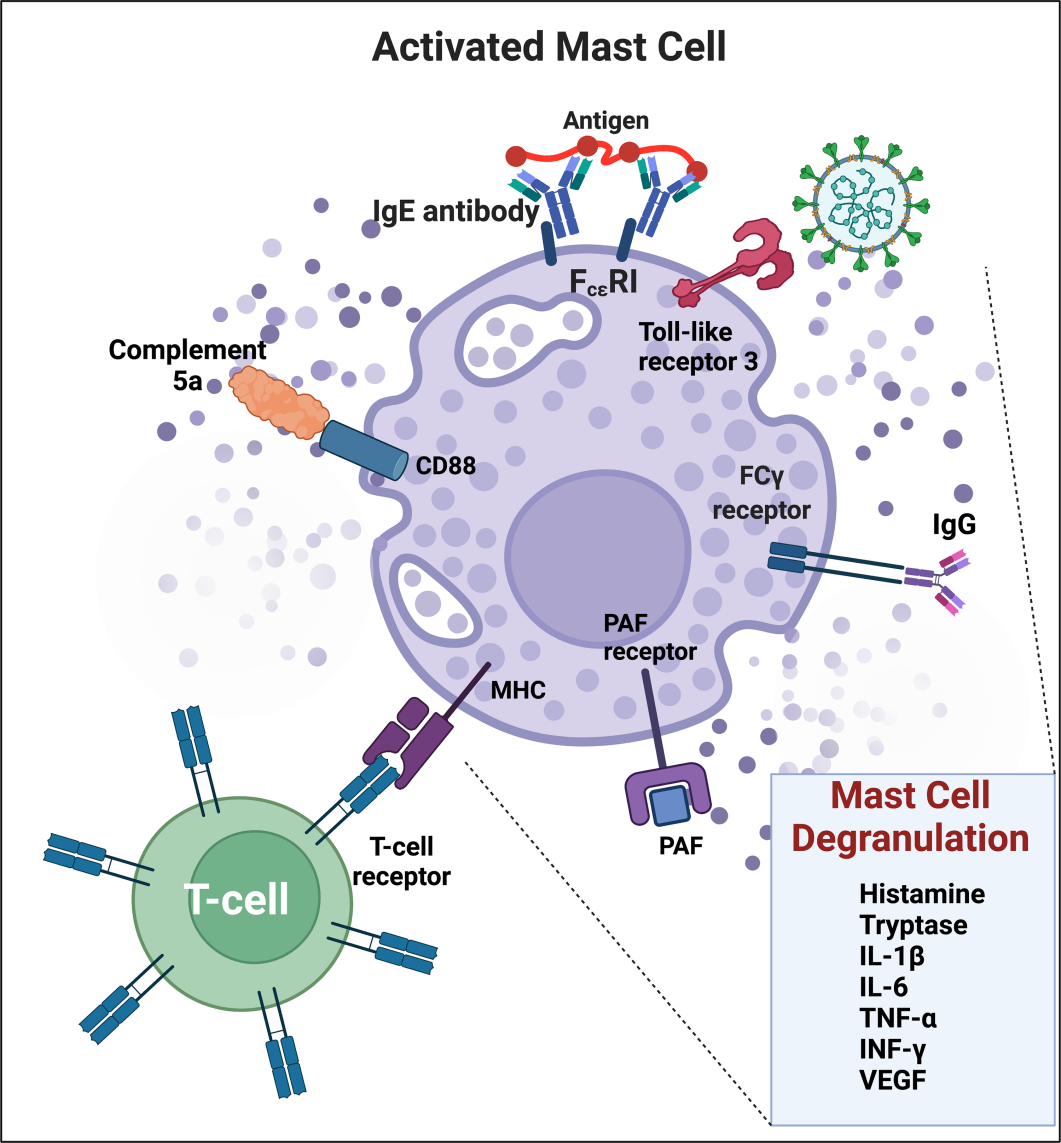
Mast cell activation during COVID-19 and the resulting inflammation and coagulation pathology via mast cell degranulation. SARS-CoV-2 can activate mast cells through TLR(3) to release proinflammatory mediators. TLR, Toll-like receptor; FcεRI, high affinity IgE receptor; FcγR, IgG receptor; PAF, platelet-activating factor; MHC, major histocompatibility complex. Adapted from [635–637]. Created with BioRender (https://biorender.com/).

Another mechanism mediating hyperinflammation is mast cell activation [621]. It occurs during acute COVID [622–624], and the symptoms [625] of those with known mast cell activation syndrome significantly mirror those of individuals with Long COVID [626,627]. The elevation of IL-6 levels, as well as proteases such as carboxypeptidase A3 and tryptase, is a hallmark of this and these are also observed in PASC patients [628]. Importantly for the present focus, mast cells are also activated during (and exacerbate) ischaemia–reperfusion injury [629–634], and the inhibition of mast cell degranulation [634] might consequently be of benefit in Long COVID. Indeed, these findings are consistent with the known beneficial effects of antihistamines in PASC [80] (see Figure 9).

## Platelet hyperactivation

**Figure 10.**
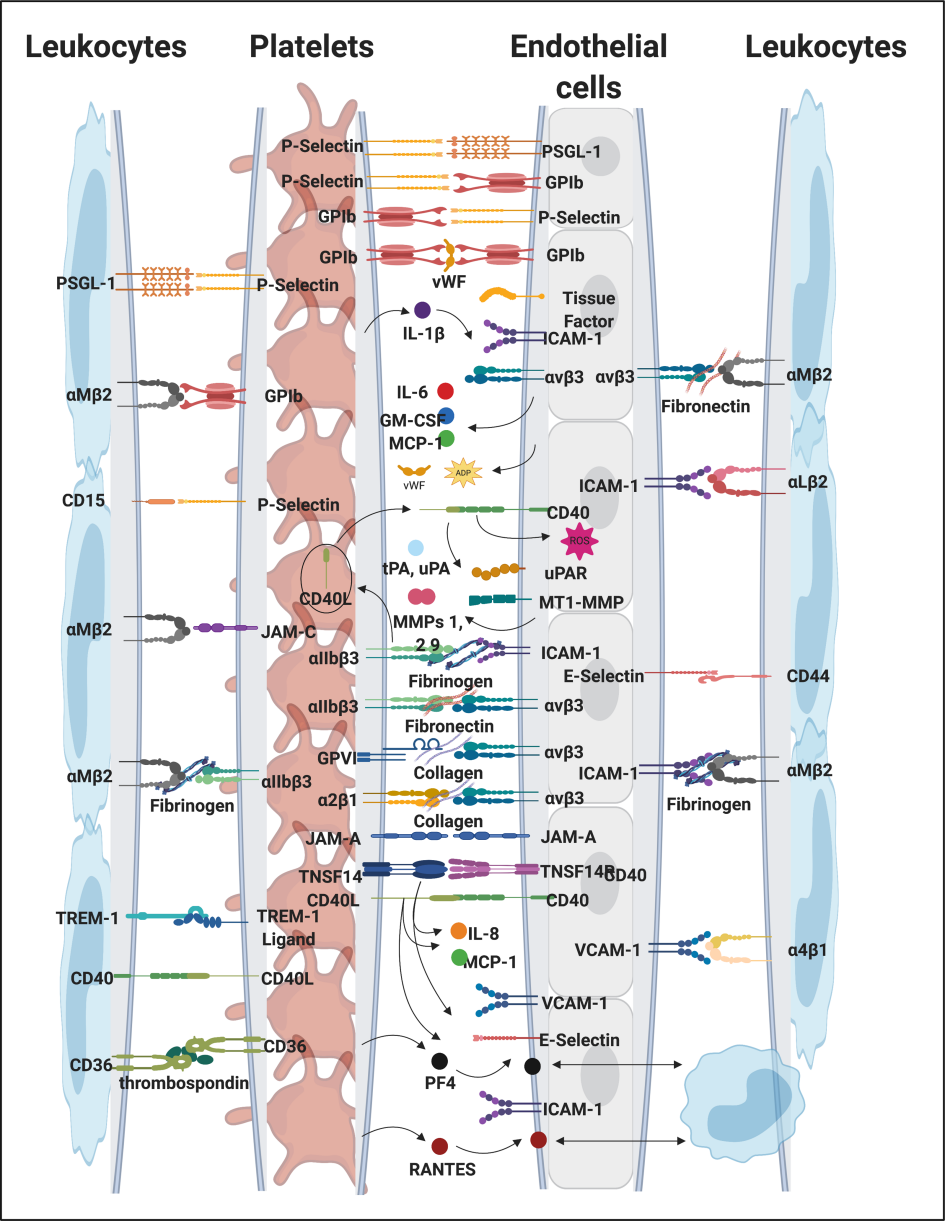
Platelet receptors and interactions with inflammatory molecules, endothelium and leucocytes during inflammation and clotting pathologies (adapted from [652]). Created with BioRender (https://biorender.com/).

**Figure 11.**
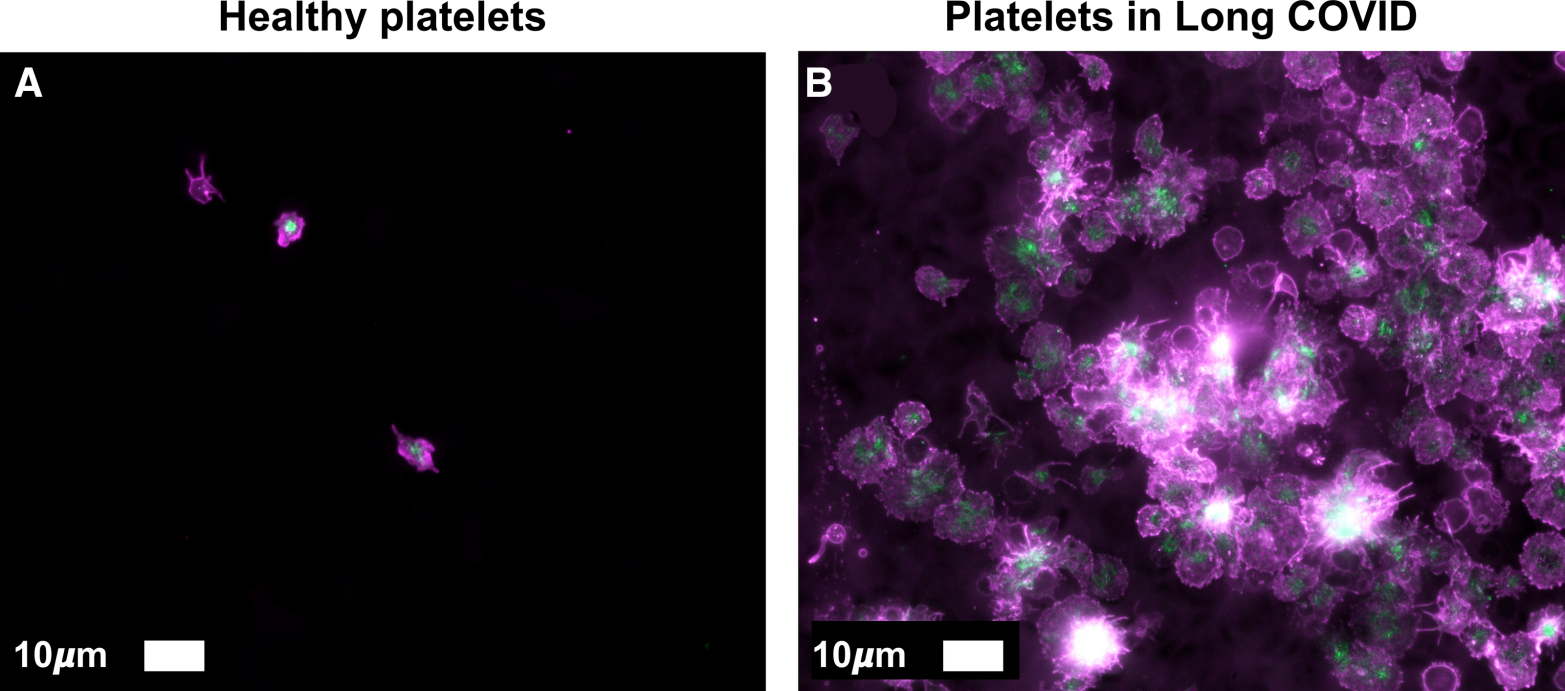
Platelet hyperactivation noted in a healthy individual (A) and an individual with Long COVID with severe platelet hyperactivation (B). Haematocrit samples were exposed to the two fluorescent markers, CD62P (PE-conjugated) (platelet surface P-selectin) (IM1759U, Beckman Coulter, Brea, CA, U.S.A.) and PAC-1 (FITC-conjugated) (340507, BD Biosciences, San Jose, CA, U.S.A.). CD62P is a marker for P-selectin that is either on the membrane of platelets or found inside them. PAC-1 identifies platelets through marking the glycoprotein IIb/IIIa (gpIIb/IIIa) on the platelet membrane. Samples were viewed using a Zeiss Axio Observer 7 fluorescent microscope with a Plan-Apochromat 63x/1.4 Oil DIC M27 objective (Carl Zeiss Microscopy, Munich, Germany). (Unpublished data; Ethics from Stellenbosch University Human Ethics Committee (HREC) number 9521.).

Platelet hyperactivation is one of the observables of both acute [638,639] and Long COVID [89], and one of its causes can certainly be oxidative stress [640], as well as circulating inflammatory molecules that may trigger platelet hyperactivation, spreading, and significant platelet clumping [86,301]. It is also part of ME/CFS [641]. Platelets have numerous receptors on their membranes that may interact with circulating inflammatory molecules, including viral and bacterial inflammagens [82,642–647]. In addition, platelets and their membrane receptors may also interact with each other, with endothelial cells and with other immune cells, forming platelet complexes. These interactions not only drive pathological clotting, but can also perpetuate endothelial damage and immunothrombosis [648,649], not only in COVID-19 as Long COVID, but also in all inflammatory diseases [647,650–660]. P-selectin is a well-known inflammatory molecule that may be found inside the granules of healthy platelets, on the membrane of the activated platelet where it acts as a binding receptor, or in circulation, as a soluble inflammatory molecule [82,301,646,661,662], Levels were raised in both participants in a small study [663]. Figure 10 shows selected platelet receptors and Figure 11 examples of platelet hyperactivation in individuals with Long COVID. Platelets are well-known for their storage of serotonin [664] and platelet factor 4 (PF4) and serotonin are stored in α- and δ-granules [665]. Circulating immune complexes may also activate platelets via receptor–receptor binding followed by the release of serotonin from platelet granules [665]. A subpopulation of platelets (the COAT-platelets) activated with collagen and thrombin express functional α-granule factor V. These COAT-platelets can bind fibrinogen, VWF, thrombospondin, fibronectin, and α2-antiplasmin [666]. In addition, COAT-platelets use serotonin conjugation to bind pro-coagulant proteins on their cell surface through a serotonin receptor [666]. As platelet serotonin is the main source of serotonin in the blood, if a significant proportion of the circulating platelet population is hyperactivated, these platelets will shed their serotonin content and it will, in addition, provide a pro-coagulant surface, allowing these platelets to form platelet complexes, bind to fibrin(ogen), and also damaged endothelial cells (see Figure 10). Platelet hyperactivation has recently also been observed in ME/CFS [352].

Interestingly, serotonin receptor antagonists reverse serotonin-mediated pulmonary vasoconstriction, lessen pulmonary platelet trapping, inhibit platelet activation and aggregation, and normalise increased respiratory drive, in severe COVID-19 [667,668]. It was also noted that there is an inverse association between serotonin antagonist medication usage and mortality in severe COVID-19 mortality [669].

## Autoimmunity

Autoimmunity is of course a chief feature of rheumatoid arthritis, and the details are in principle reasonably well understood [14,102–105,670]. It seems that autoimmunity is also a significant contributor to the after-effects of many viral infections [671], including Long COVID [672,673]. The microclots entrap a great many proteins [88], and of course any protein whose conformation is changed may present a new epitope that appears as ‘non-self’ and thus elicits autoantibodies. In addition, the nitration of proteins — as occurs during and following oxidative stress — also leads to the production of autoantibodies [674] and autoantibodies for actin lead to muscle weakness [587]. Autoantibodies are a well-known element in ME/CFS [20,98,548,675,676], and we consider they are likely to play an increasing role as the duration of Long COVID extends, since various autoantibodies share elements of epitope with the SARS-CoV-2 virus [677], and some of the viral sequences are in fact amyloidogenic themselves [678,679]. Since there is significant evidence for viral persistence during Long COVID (e.g. [680], and below), if they and autoantibodies also possess the ability to stimulate fibrin amyloid microclots, this provides one straightforward mechanism for continuing microclot persistence.

## A gut microbiome reservoir for SARS-CoV-2?

In some cases, we are aware that there seems to be a continuation of Long COVID symptoms in spite of all kinds of treatments, and one explanation involves a unusual extent of viral persistence [681], for which there is increasing evidence (e.g. [19,682]). To this end, the suggestion of Brogna et al. [683] that SARS-CoV-2 could act like a bacteriophage and use bacteria as replication hosts is of especial interest. The gut microbiome does of course contain many trillions of organisms [684,685], contributes massively to immunity and inflammation [686], provides a clear pathway between diet and health [687], and would be the most logical place for such a reservoir to persist. The composition of the microbiome is also highly influenced by all kinds of drugs (including [688] but far beyond antibiotics [689–691]). Certainly, the gut and oral [692] microbiomes are highly dysregulated in both acute [693,694] and Long COVID [19,695,696], though the extent to which susceptibility to the disease is a cause or a consequence or both (or involving attendant medications) is largely unclear [697]. This said, one study [695] showed clear predictability between the microbiome and the nature and likelihood of PASC symptoms, while another showed it tracked recovery [698]. Overall it would be astonishing if improvements in the gut microbiome were not accompanied by improvements in the symptoms of PASC, and that certain unfavourable organisms might serve as intermediate hosts for viral replication in the gut. If this is the case, a reset using antibiotics followed by pre- and pro-biotics would seem to be the correct strategy. Persistence through cell–cell viral transmission without release [699] may also occur.

## Candidate treatments: drugs

**Figure 12.**
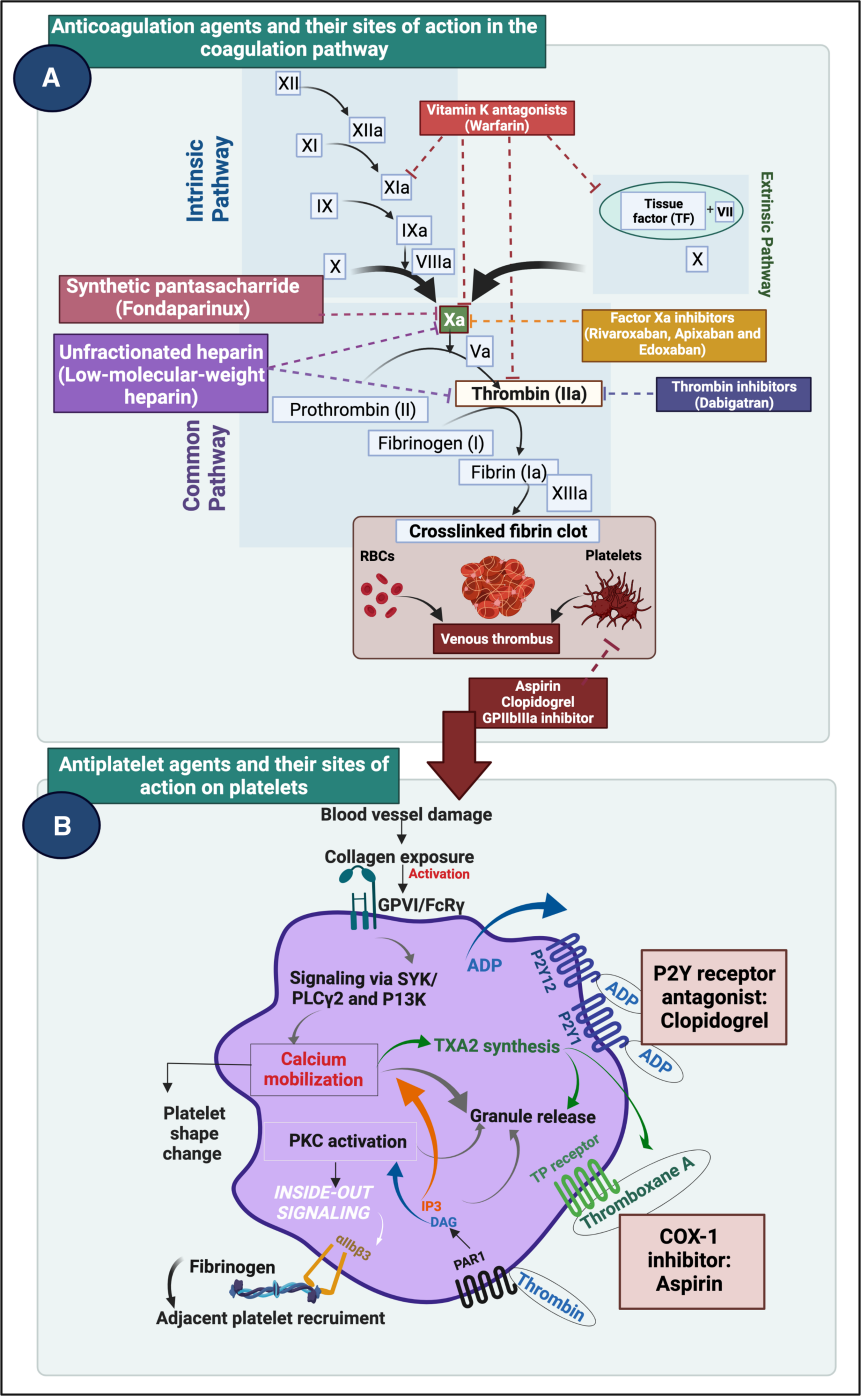
Effects of selected direct oral anticoagulants (DOAC) and dual antiplatelet therapy (DAPT) medication on clotting and platelet function. Created with BioRender (https://biorender.com/).

Based on what we have seen above and now know, it is reasonable to rehearse known pharmaceutical drugs [548] for which there is evidence of benefit for those in various stages of ME/CFS or Long COVID. Of course, the efficacy in treating rheumatoid arthritis of various small molecules (‘DMARDs’, which were in fact originally isolated as antibiotics) and anti-inflammatory molecules such as antibodies against TNF-α [700,701], is very well known, giving credence to the importance of such kinds of processes in Long COVID. Antiplatelet and anticoagulation medication may work on different parts of the clotting cascade by blocking platelet activation or by preventing new clots from forming by blocking the enzymatic pathway [86,702–704]. See Figure 12 that shows selected direct oral anticoagulants (DOAC) and dual antiplatelet therapy (DAPT) medication on clotting and platelet function.

**Figure 13.**
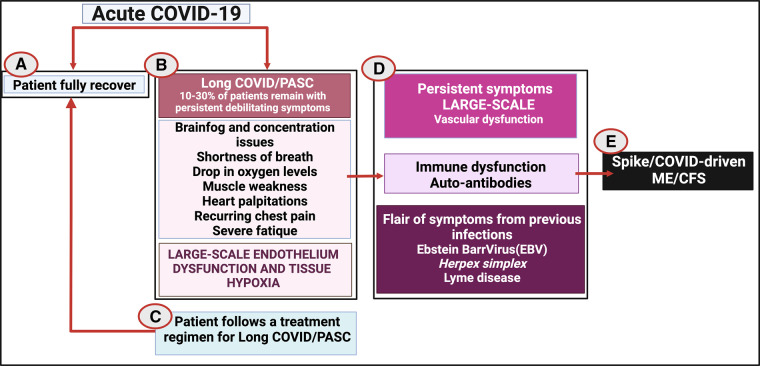
Possible Long COVID disease progression if left untreated. Disease/recovery progression developing the acute disease to (**A**) full recovery; (**B**) developing Long COVID/PASC; (**C**) treatment for Long COVID/PASC and subsequent recovery; (**D**) no treatment or positive treatment outcomes and the eventual development of spike/COVID-driven ME/CFS. Created with BioRender (https://biorender.com/).

If it is confirmed that individuals with Long COVID do indeed have platelet hyperactivation and microclot presence in their circulation, and these pathologies are not sufficiently treated, we hypothesise that a few scenarios might then develop (see Figure 13):

- Patients recover spontaneously where their fibrinolytic system returns to healthy clotting and lysis cycles.
- Patients do not spontaneously recover, but instead develop a persistent hypercoagulable state with the persistent triggering of hyperactivated platelets and persistent endotheliitis, that may lead to more widespread endothelial damage.
- Microclots continuously entrap inflammatory molecules and will eventually cause immune dysfunction and even autoimmunity.
- Some individuals, who previously might have suffered from EBV, *Herpes simplex* virus or Lyme disease might suffer from a flare of those original symptoms, caused by reinfection of even by the vaccine.
- In some individuals the persistent microclots, and widespread endothelial pathology may culminate in eventually COVID triggering ‘spike/COVID-driven ME/CFS’.

## Antivirals

Given that viruses tend to persist (whether in dormant or more active forms) [680], antivirals seem like a logical component of any therapy for ME/CFS [705], though the evidence of strong benefits of any individual drug is still weak [706]. As with any complex system, it is likely that multiple targets will need to be modulated simultaneously [548]. In the case of Long COVID, it is still too early to know what benefits, if any, will come from the deployment of antivirals (which are often quite toxic); the antiparasitic drug ivermectin was advocated as an antiviral in some quarters, but seems not to be effective [707].

## Anticoagulants, platelet inhibitors, and the triple treatment

### Heparin and Fondaparinux

Heparin is a well-known regulator of the coagulation cascade that is also a potent inhibitor of angiogenesis [708] (see Figure 12). Heparin, therefore, directly modulates the coagulation cascade and is an excellent anticoagulant in diseases where hypercoagulation is prevalent. There are two types of heparins are that are widely used in prophylactically and as treatment regimes: unfractionated heparin (UFH) and low molecular mass heparin (LMWH). UFH can bind to antithrombin (SERPINC1) via a pentasaccharide, catalysing the inactivation of thrombin and other clotting factors. UFH also binds endothelial cells, PF4, and platelets [709]. Antithrombin is an essential regulator of the coagulation cascade and of proteolytic activity, as it acts to inactivate several enzymes of the coagulation cascade. It is well-known to inhibit thrombin and multiple other coagulation factors e.g. FIXa, Xa, XIa, and XIIa. It acts in both the intrinsic and extrinsic pathways [710]. The Heparin of choice is currently LMWH, as it lacks the nonspecific binding affinities of UFH, and has more predictable pharmacokinetic and pharmacodynamic properties [709]. Fondaparinux (a synthetic heparin pentasaccharide with a sequence identical with that found in anticoagulant heparin) is a well-known antithrombotic agent for the prevention and treatment of VTE and in ischaemic heart disease without significant bleeding risk [711]. It has also been suggested that Fondaparinux should be used in the treatment of COVID-19 coagulopathies [712]. As well as its role as an anticoagulant in decreasing mortality with acute COVID-19 [713] it was recognised early in the piece [714] that heparin binds to the SARS-CoV-2 spike protein and can thus inhibit its entry into cells [715]. The usefulness of antithrombotics was also recently discussed in a paper that investigated outcomes of antithrombotic use in patients with atrial fibrillation who subsequently developed COVID-19 [716]. It was found that individuals that were on antithrombotic therapies before they developed COVID-19 were less likely to die from the infection.

In 2020, Viecca and co-workers reported on a single-centre, investigator initiated, proof of concept, case-control study, conducted in Italy. Specifically, the effects of antiplatelet therapy on arterial oxygenation and clinical outcomes in patients with severe COVID-19 with hypercoagulability were investigated in a phase IIb trial (NCT04368377) [717]. Patients received 25 μg/kg body weight tirofiban as bolus infusion, followed by a continuous infusion of 0.15 μg/kg body weight per minute for 48 h. Before tirofiban, patients received acetylsalicylic acid 250 mg infusion and oral clopidogrel 300 mg; both were continued at a dose of 75 mg daily for 30 days. Fondaparinux 2.5 mg/day sub-cutaneous was given for the duration of the hospital stay. The study found that antiplatelet therapy might be effective in improving the ventilation and perfusion ratio in COVID-19 patients with severe respiratory failure and that the therapy prevented clot formation in lung capillary vessels. A recent 2022 JAMA paper [718] discussed the outcome of a trial [719] where moderately ill hospitalised patients with COVID-19, who were given the P2Y12 inhibitor Ticagrelor (in addition to therapeutic doses of heparin) did not improve their health outcome. Data were compared with a group of patients who received only a therapeutic dose of heparin.

### Biologics as anti-inflammatories

Inflammation is strongly associated with (and virtually defined as) the production of inflammatory cytokines such as IL-6 and TNF-α (see Figure 9). Any means of lowering either the cause or such effects of inflammation is likely to be beneficial, and so it has proven, with the anti-TNF-α antibodies such as adalimumab (Humira), etanercept (Enbrel), and infliximab (Remicade) demonstrating huge benefits against rheumatoid arthritis, as well as other automimmune diseases. As biosimilars begin to come in (e.g. [720–722]), the substantial costs of the originals [723] may be expected to fall considerably. While there are grounds for optimism that these drugs can assist in the treatment of both acute [724–726] and Long COVID [727], it is too early yet to have the numbers to tell if they will [728]. However, one might have expected them to have been trialled more often in patients with ME/CFS [729], where a variety of inflammatory cytokine levels are also raised [730–732]. Consequently, we consider that such drugs might have considerable benefits in the treatment of both ME/CFS and Long COVID. We note in particular that RA patients taking anti-TNF-α therapies experienced major improvements in their fatigue symptoms [733].

### Other drugs

Colchicine has long been used in various inflammatory diseases, and has shown promise in acute COVID-19 [734]. Other drugs being studied for use in ME/CFS or Long COVID include metformin [735–737], fenofibrate (which is somewhat protective against reperfusion injury [738,739]), and low-dose naltrexone [740–742]. While we are no experts, and the mechanisms are normally not well understood [743], but as a segue to the following section on nutraceuticals, we also note the effective use of certain traditional Chinese and other traditional medicines in acute COVID-19 [744–749] and their potential for use in Long COVID [748–750]. This seems like an area well worth further study by those qualified to do so.

### Candidate treatments: nutraceuticals

Given the mismatch between the time taken to get a new drug approved and the urgency of the long COVID pandemic, both the literature and social media have turned to the use of nutraceuticals [751–753], at least for treating the symptoms of ME/CFS, Long COVID, and related disorders. It is inconceivable [754] that any single one will work for all individuals, but we consider it worthwhile to rehearse the kinds of nutraceuticals and less mainstream approaches that people have tried in the past for chronic, inflammatory diseases, particularly those whose efficacy may provide evidence the role of ischaemia and I–R injury. This allows us to assess them within the framework of the significance of microclots and oxidative stress caused by chronic ischaemia–reperfusion events that we consider to be a substantial part of these syndromes. Such agents include anti-clotting agents, iron chelators, and antioxidants more generally [3,540,545,755–758] (although the latter two are heavily bound up with each other [106]).

### N-acetyl cysteine

N-acetyl cysteine is a well established and widely used antioxidant and anti-inflammatory molecule [759–762], which acts both to increase the intracellular levels of glutathione and to decreases the downstream activities of NF-κB. It has shown benefits in rheumatoid arthritis [763–765], in various viral diseases [766], and in abating the cytokine storm in acute COVID [767,768].

### Curcumin

Curcumin is a polyphenol antioxidant and the active constituent (and main colouring agent) of the spice turmeric. It has shown benefits in a variety of diseases involving oxidative stress [542,769–774], including ME/CFS [234,775], rheumatoid arthritis [776–780], and acute COVID-19 [781–783]. While curcumin is a well-established antioxidant, it should be noted that it also may have antiplatelet activities [784–786]. In consequence, it is contra-indicated (i.e. not recommended for) use with other anticoagulants or blood thinners.

### Ergothioneine

Ergothioneine is a major antioxidant [787–794] considered sufficiently important to the host during evolution that a natural transporter (SLC22A4 in humans) has been selected to ensure its uptake [795–797]. As we originally proposed [798], its anti-inflammatory potency has been established in a model of pre-eclampsia [799]. It is strongly protective against diseases of oxidative stress affecting the heart [793,800], liver [801,802], kidney [802], CNS [803,804], and other tissues (e.g. [794,805]. Consequently, it has been proposed as a suitable antioxidant for use in COVID-19 amelioration [806]. Most pertinently to the present analysis, it has been shown to be of benefit in preventing ischaemia–reperfusion injury [794,801,807], and so could have real value in chronic diseases that exhibit it. It is not easily obtained in pure form (though biotechnological processes are starting to make it [808–817]), but its availability via mushrooms can provide a convenient supply [818–820]. Indeed mushrooms themselves have been shown to be highly protective against mild cognitive impairment [821], as well as other diseases involving oxidative stress [787], and are themselves under consideration and trial as anti-COVID-19 agents [822,823].

### Flavonoids

Flavonoids of various kinds, often referred to as polyphenols [824], are widely recognised as antioxidants with the potential in ameliorating inflammatory diseases involving oxidative stress (e.g. [606,825–832]). Since much of their bioavailability depends on suitable transporters [827,833] it would seem wise to use a cocktail. They have shown benefits in rheumatoid arthritis [778,826,834–842], in ME/CFS [843–845], and even activity against coronaviruses [846] such as the SARS-CoV-2 responsible for acute COVID-19 [847–858] and Long COVID [859].

### Iron chelation

As noted above, and reviewed e.g. in [15,106], free iron can contribute massively to oxidative stress. Ferritin is the main *intra*cellular storage molecule for iron, and serum ferritin is a marker of cell death [149]; it is, therefore, unsurprising that it is raised massively in acute SARS-CoV-2 infection, and especially so in non-survivors [512,860–866]. Consequently, molecules that chelate iron fully (i.e. via all six of its chelation sites, see above) can serve to relieve oxidative stress [862,865,867–870] and inhibit SARS-CoV-2 effects [871]. Other nutraceutical iron chelators such as green tea catechins (epigallocatechin-3-gallate, also a polyphenol) [872–881] were discussed in detail previously [106].

### Lactoferrin

As commented in the previous section, iron dysregulation is another important element of all these chronic, inflammatory diseases, both through its behaviour in catalysing ROS formation [106,266] and hypercoagulation [326], and its ability to awaken dormant microbes [15]. Since it binds iron effectively, as well as various cell surface receptors used by SARS-CoV-2, oral lactoferrin has been proposed as a suitable treatment for (and indeed preventive of) COVID-19 [372,868,869,882–894].

### Magnesium

Although blood levels of magnesium ions are more-or-less tightly regulated, ‘magnesium’ was experimentally one of the earliest substances that we found to inhibit fibrin amyloid microclotting (then known as dense matted deposit formation) [374]. Intriguingly, populations exhibiting low magnesium ion levels were found to be more susceptible to COVID-19 [895], and magnesium supplementation has shown benefits in SARS-CoV-2 therapy [896], ME/CFS [93,897], and in maintaining endothelial cell function [898].

### Melatonin

Melatonin is a natural small molecule (a tryptophan derivative) produced in the pineal gland and involved in the induction of sleep [899]; it has been strongly promoted for its anti-oxidative and anti-nitrosative properties [900–902], including in COVID [903–911], and I–R injury [912], and is a useful ligand for free iron [106].

### Thrombolytics: nattokinase, serrapeptase, lumbrokinase, and bromleain

Clots are normally removed by plasmin, a serine protease, but some clots (such as fibrin amyloid microclots) contain antiplasmin compounds [88]; the plasma of acute COVID patients also contains anti-thrombolytic compounds [913–915], despite raised levels of tissue plasminogen activator (tPA) [915]. However, a variety of other enzymes have thrombolytic activity [916–919]. Considered less potent and safer than post-stroke ‘clotbusters’ such as tPA [920], nattokinase is a fibrinolytic [921–923] (and amyloid-degrading [924]), orally available despite having to pass through the gut wall [925–931], safe [932,933], serine protease enzyme from *Bacillus subtilis* (an organism which may itself be of value [934]). It is found naturally in the Japanese fermented food nattō [751,928,935–939], (which is also a source of vitamin K, and has antiviral properties directly [940]). Its structure is known [941,942], and it may also be produced recombinantly [943–950]. It also has antiplatelet [951], anti-inflammatory [952], and anti-hypertensive [953] properties, and along with pycnogenol [954] was active in preventing DVT on longhaul flights [955]. Serrapeptase (serratiopeptidase) [934,956–959] has a similar activity (as well as others such as mucolytic behaviour [958]) and comes from a *Serratia marcescens* strain that originates in the guts of silkworms, where it has a natural role in helping the worms emerge from their cocoons. Lumbrokinases are another orally active [960] set of fibrinolytic enzymes that have been found in earthworms [961–965], and may also be produced recombinantly [966,967]; they may also have some tPA activity [965]. Each has been proposed as of value in acute and/or Long COVID treatment [751,958,968]. With two (positive) exceptions [968,969], and another planned [970], randomised controlled trials are awaited. In the plant kingdom, bromelain (from pineapples) is a cysteine protease found in pineapple tissue [971–974]. Multiple effects imply its utility in preventing or treating SARS-CoV-2 infection and acute COVID-19 [975–978]. Given the significance of fibrin amyloid microclots; however, it would seem of value (i) to stress the importance of quality control in nutraceutical production and (ii) to assess the comparative activities of these enzymes in removing fibrin amyloid microclots *in vitro*.

### Vitamins

There is little doubt that many individuals may be deficient in their dietary supply of one or more vitamins [979]. In particular, there is evidence for the importance of vitamins B_12_ [980–983], D_3_ [906,983–986], and K_2_ [987–990] in benefitting outcome from acute COVID. However, in some cases where ‘vitamin D’ is not explicitly measured as D_3_ the evidence is equivocal [991–993]. As mentioned above, vitamin C [994] is to be recommended only if one is sure that free or poorly liganded iron is absent. Niacin (vitamin B_3_) may also be of value in a tapered-up strategy, since NAD+ levels are known to be lowered in COVID-19 [995] and other viral infections [996]; its metabolite 1-methylnicotinamide has also shown promise in Long COVID [997].

Overall, there is a considerable body of evidence that nutraceuticals active as antioxidants, iron chelators, or fibrinolytics, as well as other targets, may be of benefit in the sets of diseases under consideration here, consistent with the role of ischaemia–reperfusion injury therein.

## Other, non-pharmacological methods

### H.E.L.P.: apheresis

Apheresis refers to the specific extracorporeal removal of particular substances from blood, such as lipids [998,999]. Originally introduced for the lowering of low-density lipoproteins (LDLs) (e.g. [1000,1001]), Heparin-mediated LDL precipitation (H.E.L.P.) apheresis has come to the fore as a means for assisting Long COVID patients, since it too seems to remove fibrin amyloid microclots with high efficiency. Alternative aphereses [1002,1003] also be of value.

### Hyperbaric oxygen therapy

If ischaemia is an important part of Long COVID, then preventing it (while not mimicking reperfusion) should be of value [1004,1005]. To this end, hyperbaric oxygen therapy [1006–1008] has been recommended, and in some cases found useful in rheumatoid arthritis [1009–1011], acute [1012,1013] and Long COVID [1006,1014]. An alternative involves O_2_ nanobubbles [1015].

### Concluding remarks

**Figure 14.**
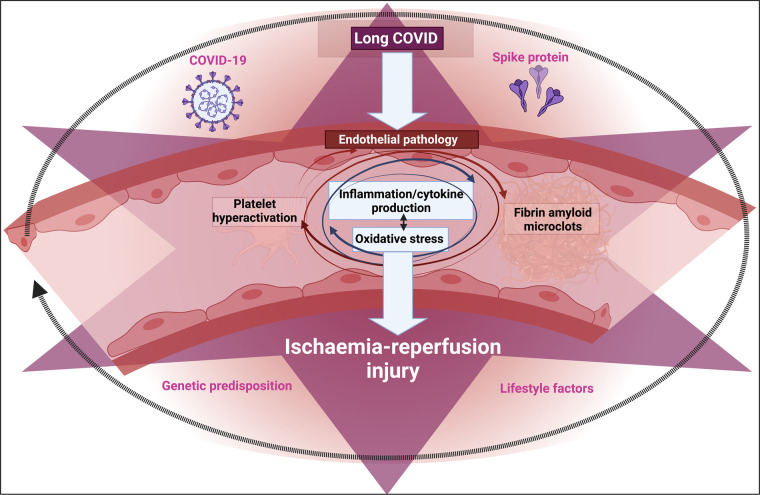
Some of the major elements of Long COVID and related diseases that we have highlighted here. They illustrate the complexity of ischaemia–reperfusion injury driven by platelet hyperactivation, fibrin amyloid microclots, circulating inflammatory molecules/cytokine production. Created with BioRender (https://biorender.com/).

We have sought to bring together what is the very considerable evidence that many of the features of Long COVID resemble those observed in ME/CFS, and to some degree those in rheumatoid arthritis, and that a common denominator may be fibrin amyloid microclots and damaged cell structures that block up capillaries and can consequently lead to oxidative stress and ischaemia–reperfusion injury (Figure 14).

Many of the predicted sequelae have indeed been observed, and thus provide evidence for this general mechanism of chronic illness. If the analysis is correct it implies that considerable therapeutic benefits are to be had from strategies that inhibit the formation of the microclots and that act — especially the use of antioxidants — to diminish the effects of ROS by mopping them up. A variety of further predictions remain to be tested, but we hope that we have set them out clearly enough to enable others to do so.

## Consent for publication

All authors approved the submission of the paper.

## Abbreviations

CR1: complement receptor 1
DVT: deep vein thrombosis
EBV: Epstein–Barr virus
ESR: erythrocyte sedimentation rates
I–R: ischaemia–reperfusion
LDLs: low-density lipoproteins
LMWH: low molecular mass heparin
PE: pulmonary embolism
PEM: post-exertional malaise
PF4: platelet factor 4
RDW: red cell distribution width
ROS: reactive oxygen species
TBARS: thiobarbituric acid reactive substances
tPA: tissue plasminogen activator
UFH: unfractionated heparin
VTE: venous thromboembolism
VWF: von Willebrand factor
